# Integration of genetic and physical maps of the *Primula vulgaris S* locus and localization by chromosome *in situ* hybridization

**DOI:** 10.1111/nph.13373

**Published:** 2015-04-09

**Authors:** Jinhong Li, Margaret A. Webster, Jonathan Wright, Jonathan M. Cocker, Matthew C. Smith, Farah Badakshi, Pat Heslop‐Harrison, Philip M. Gilmartin

**Affiliations:** ^1^ School of Biological Sciences University of East Anglia Norwich Research Park Norwich NR4 7TJ UK; ^2^ John Innes Centre Norwich Research Park Norwich NR4 7UH UK; ^3^ The Genome Analysis Centre Norwich, Research Park Norwich NR4 7UH UK; ^4^ School of Biological Sciences Durham University Durham DH1 3LE UK; ^5^ Department of Biology University of Leicester Leicester LE1 7RH UK

**Keywords:** chromosome *in situ*, genetic map, heterostyly, *Primula vulgaris*, *S* locus

## Abstract

- Heteromorphic flower development in *Primula* is controlled by the *S* locus. The *S* locus genes, which control anther position, pistil length and pollen size in pin and thrum flowers, have not yet been characterized. We have integrated *S*‐linked genes, marker sequences and mutant phenotypes to create a map of the *P. vulgaris S* locus region that will facilitate the identification of key *S* locus genes. We have generated, sequenced and annotated BAC sequences spanning the *S* locus, and identified its chromosomal location.
- We have employed a combination of classical genetics and three‐point crosses with molecular genetic analysis of recombinants to generate the map. We have characterized this region by Illumina sequencing and bioinformatic analysis, together with chromosome *in situ* hybridization.
- We present an integrated genetic and physical map across the *P. vulgaris S* locus flanked by phenotypic and DNA sequence markers. BAC contigs encompass a 1.5‐Mb genomic region with 1 Mb of sequence containing 82 *S*‐linked genes anchored to overlapping BACs. The *S* locus is located close to the centromere of the largest metacentric chromosome pair.
- These data will facilitate the identification of the genes that orchestrate heterostyly in *Primula* and enable evolutionary analyses of the *S* locus.

## Introduction

Heterostyly is found in over 160 genera across 24 families (Ganders, 1979), suggesting a polyphyletic origin (Barrett, 1990). Despite different origins and therefore potentially different mechanisms, the locus controlling heterostyly is uniformly known as the *S* locus (Lewis & Jones, 1993), and is diallelic in *Primula vulgaris* (Bateson & Gregory, 1905), *Turnera subulata* (Shore & Barrett, 1985), *Fagopyrum esculentum* (Garber & Quisenberry, 1927) and *Linum grandiflorum* (Ushijima *et al*., 2012). Molecular genetic studies on floral heteromorphy have focused on species from four families: Turneraceae (*T. subulata*) (Athanasiou *et al*., 2003; Labonne *et al*., 2008, 2009; Labonne & Shore, 2011); Polygonaceae (*F. esculentum*) (Wang *et al*., 2005; Yasui *et al*., 2008, 2012); Primulaceae (*P. vulgaris*) (Manfield *et al*., 2005; McCubbin *et al*., 2006; Li *et al*., 2007, 2008, 2010; Cocker *et al*., 2015); and Linaceae (*L. grandiflorum*) (Ushijima *et al*., 2012).

In *Primula*, flowers have either a long style, low anthers and small pollen, or a short style, high anthers and large pollen (Darwin, 1862). *Fagopyrum esculentum* (Yasui *et al*., 2012) and *T. subulata* (Labonne & Shore, 2011) also have long‐styled and short‐styled flowers. In these species, the anther filament length determines the height of the anthers in different floral morphs; in *Primula*, it is the point of attachment to the corolla tube that differs between pin and thrum flowers (Darwin, 1862; Webster & Gilmartin, 2003). *Linum grandifolium* shows stigma–height dimorphism with flowers that differ in stigma height, but not the position of the anthers (Darwin, 1863; Barrett, 2010; Ushijima *et al*., 2012).

In *Turnera,* progress towards the identification of the *S* locus includes a high‐resolution genetic map (Labonne *et al*., 2009) and the identification of deletion mutants (Labonne *et al*., 2010). This approach enabled the assembly of three BAC contigs spanning the *S* locus which, in combination with deletion mutants, enabled the positional cloning of the recessive *s* allele in *T*. *subulata* (Labonne & Shore, 2011). Although the key genes have not yet been described, these studies represent a significant step towards the identification of the molecular mechanisms of floral heteromorphy in this species. Similar map‐based approaches have been used in *F*. *esculentum* (Yasui *et al*., 2004, 2008; Konishi *et al*., 2006), where next‐generation sequencing and *in silico* analysis have identified a candidate *S* locus gene, *S‐ELF3* (Yasui *et al*., 2012). The analysis of a short‐styled chromosome deletion mutant, which produces long‐styled flowers, revealed that *S‐ELF3* had been lost in the deletion. Although the large deletion may contain other genes, mutations in *S‐ELF3* in the other homomorphic cultivars suggest that *S‐ELF3* is a candidate regulator of heteromorphic flower development in *Fagopyrum*. Approaches to study floral heteromorphy in *L. grandiflorum* (Ushijima *et al*., 2012) used a combination of suppressive subtractive hybridization and two‐dimensional‐polyacrylamide gel electrophoresis (2D‐PAGE) analysis to reveal 12 floral morph‐related genes. Four genes implicated in the control of style length (Ushijima *et al*., 2012) include a Myb transcription factor, *LgMYB21*, which, when constitutively overexpressed in *Arabidopsis*, reduces style length and anther height.

Building on the work of Darwin, early studies on the genetics and control of heterostyly in *Primula* revealed the dominance of the thrum phenotype and defined *S* and *s* alleles (Bateson & Gregory, 1905; Gregory, 1911). Subsequent analysis (Ernst, 1925, 1936b; Pellow, 1928; Haldane, 1933; Dowrick, 1956; Lewis & Jones, 1993) defined three diallelic genes, *G/g*,* P/p* and *A/a*, at the *S* locus with thrums heterozygous *GPA/gpa* and pins homozygous recessive *gpa/gpa*. The rare occurrence of homostyles was predicted to arise via mutation (Ernst, 1928b, 1936a), and subsequently interpreted as a result of crossovers within the *S* locus gene cluster (Dowrick, 1956; Lewis & Jones, 1993). Subsequent studies expanded the linkage group to include genes involved in pollen size dominance (Kurian & Richards, 1997) and pollen and style self‐incompatibility behaviour (Dowrick, 1956; Lewis & Jones, 1993; Richards, 1997).

Early reports of *S*‐linked genes not involved in heterostyly include *Hose in Hose* (Ernst, 1928a, 1942) and four loci in *P. sinensis: magenta* (*b*), *red stigma* (*g*), *red leaf back* (*l*) and *double* (*x*) (De Winton, 1928; De Winton & Haldane, 1935); these plants are no longer available. More recently, quantitative trait locus (QTL) analysis of floral morphology in *P. sieboldii* provided a genome‐wide linkage map with four markers within 1 cM of the *S* locus (Yoshida *et al*., 2011). We have characterized previously *Hose in H*ose (Webster & Grant, 1990), *sepaloid* (Li *et al*., 2008) and *Oakleaf* (Webster, 2005; Cocker *et al*., 2015) as *S*‐linked phenotypes, and identified *S* locus markers by random amplified polymorphic DNA (RAPD) analysis (Manfield *et al*., 2005), fluorescent differential display (Li *et al*., 2007) and analysis of *Hose in Hose* (Li *et al*., 2010). Here, we combine these studies into an integrated genetic map of the *S* locus; we describe the assembly and sequence of a BAC contig spanning the region, and use *in situ* hybridization to define the chromosomal location of the *Primula S* locus.

## Materials and Methods

### Growth of plants and genetic crosses

The plants used in this study are cultivated varieties of *Primula vulgaris* Huds*. Primula vulgaris* cv Blue Jeans was obtained from Thompson and Morgan (http://www.thompson-morgan.com/). *Hose in Hose* (Gerard, 1597; Webster & Grant, 1990)*, sepaloid* (Webster, 2005; Li *et al*., 2008) and *Oakleaf* (Webster, 2005; Cocker *et al*., 2015) from the National Collection of *Primula*, British Floral Variants, maintained by Margaret Webster, were used to generate parental genotypes for three‐point crosses by pollination under insect‐free conditions, with the plants grown as described previously (Webster & Gilmartin, 2006).

### Southern analysis

Genomic DNA was isolated from *P. vulgaris* leaves by a Nucleon Phyto‐Pure Genomic DNA Extraction kit (GE‐LifeSciences, Little Chalfont, Buckinghamshire, UK; www.gelifesciences.com) according to the manufacturer's instructions, and Southern analysis was performed as described previously (Manfield *et al*., 2005; Li *et al*., 2010).

### Chromosome *in situ* hybridization

BACs were labelled with biotin‐dUTP or digoxigenin‐dUTP and detected with fluorescein or Alexa‐594 conjugates. *In situ* hybridization to root‐tip metaphase chromosomes, detection, counterstaining with 4′,6‐diamidino‐2‐phenylindole (DAPI), microscopy and imaging were performed as described previously (Heslop Harrison & Schwarzacher, 2002). Unlabelled *P. vulgaris* DNA (1 μg, 25 × probe amount) was added to each slide (40 μl) before hybridization to limit the hybridization of repetitive probe sequences. After hybridization, the most stringent wash in 0.05 × saline sodium citrate (SSC) at 43°C corresponded to a hybridization stringency of 78% (high‐stringency hybridization), and in 1 × SSC at 42°C to low‐stringency hybridization.

### BAC sequencing, assembly and annotation

BAC library construction and screening have been described previously (Li *et al*., 2011). Seven of 42 BACs were sequenced individually by 454GSFLX at the Centre for Genomic Research (Liverpool, UK); four were sequenced as HiSeq2000 paired‐end reads at The Genome Analysis Centre (Norwich, UK). The remaining BACs were sequenced in two pools of 19 and 18 by 454GSFLX (Liverpool, UK). Five of the BACs sequenced individually were also included in the pools. To facilitate BAC contig assembly, we used a draft genome assembly of Illumina paired‐end reads from thrum genomic DNA (83× coverage), scaffolded with a 9‐kb thrum genomic DNA mate‐pair library (32× coverage). The assembly was curated using The Genome Analysis Centre (TGAC) browser http://www.tgac.ac.uk/tgac-browser/. Contig assembly and annotation details are given in Supporting Information Methods S1. All sequences have been deposited at the National Center for Biotechnology Information (NCBI) under Bioproject number PRJEB7311. BAC contigs and annotations are available at http://browser.tgac.ac.uk/primula_vulgaris_slocus/.

## Results

### Analysis of allelic variants of *S locus‐linked* genes

**Figure 1:**
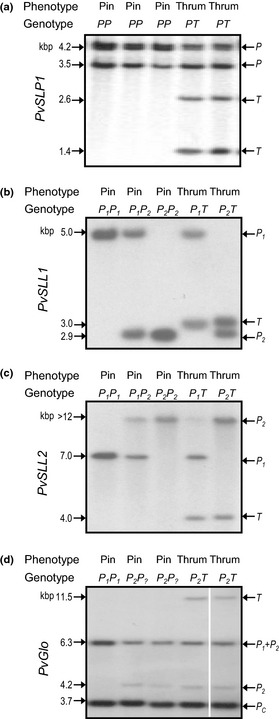
Allelic variation of *S* locus‐linked sequences in *Primula vulgaris* cv Blue Jeans. Autoradiographs following gel blot analysis of pin and thrum genomic DNA. The phenotypes of pin and thrum individuals are indicated. The genotypes of plants in relation to pin alleles (P) and thrum alleles (T) are shown and refer to the specific locus. Allele‐specific restriction fragment length polymorphism (RFLP) bands are indicted on the right and sizes in kbp on the left. Where pin alleles can be distinguished, these are indicated as P_1_ and P_2_; where unknown, as P_?_. (a) *Kpn*I‐digested genomic DNA with *PvSLP1* as probe. (b) *Hind* III‐digested genomic DNA with *PvSLL1* as probe. (c) *Xba*I*‐*digested genomic DNA with *PvSLL2* as probe. (d) *Xba*I‐digested genomic DNA with *PvGlo* as probe (lane 5 has been positioned next to lanes 1–4 to maintain sample order with parts (a–c), all five lanes are from the same gel). [Correction added after online publication 9 April 2015: panel (d) amended to include missing white line between lanes 4 and 5.]

We have identified previously four *S*‐linked markers in *P. vulgaris* cv Blue Jeans. *PvSLP1* was identified by RAPD analysis (Manfield *et al*., 2005), *PvSLL1* and *PvSLL2* by fluorescent differential display (Li *et al*., 2007) and *PvGlo* as *Hose in Hose* (Li *et al*., 2010). In each case, markers were analysed independently in different F_1_ and F_2_ individuals. Here, we show an integrated analysis of these markers in the same five plants (Fig. 1). Figure 1(a) shows restriction fragment length polymorphisms (RFLPs) for *PvSLP1*. The 4.2‐ and 3.5‐kb bands are common to pins and thrums; the 2.6‐ and 1.4‐kb bands are thrum specific (Manfield *et al*., 2005). RFLP analysis of the same five plants for *PvSLL1* (Li *et al*., 2007) (Fig. 1b) reveals two different pin alleles defined by 5.0‐kb (*P*_*1*_) and 2.9‐kb (*P*_*2*_) bands; a 3.0‐kb band (*T*) represents the thrum allele. A *P*_*1*_ *P*_*1*_ homozygote, a *P*_*1*_ *P*_*2*_ heterozygote and a *P*_*2*_ *P*_*2*_ homozygote with respect to *PvSLL1* are shown. The two thrum plants have genotypes *P*_*1*_ *T* and *P*_*2*_ *T* (Fig. 1b). RFLP analysis of *PvSLL2* (Li *et al*., 2007) using the same plants reveals two pin alleles distinguished by 7.0‐kb (*P*_*1*_) and > 12‐kb (*P*_*2*_) bands; a 4.0‐kb band represents the thrum allele (*T*) (Fig. 1c). This example shows individuals with *P*_*1*_ *P*_*1*_, *P*_*1*_ *P*_*2*_, *P*_*2*_ *P*_*2*_, *P*_*1*_ *T* and *P*_*2*_ *T* genotypes with respect to *PvSLL2*.

Figure 1(d) illustrates the RFLP profile obtained with *PvGlo* (Li *et al*., 2010) as a probe using the same five plants. A 3.7‐kb hybridization band (*P*_*c*_) is common to pin and thrum alleles of *PvGlo*. A 6.3‐kb band, also found in pins and thrums, derives from both *P*_*1*_ and *P*_*2*_ alleles, and is designated *P*_*1*_ *+ P*_*2*_. The plant in lane 1 lacks the 4.2‐kb *P*_*2*_ allele, but shows a stronger signal for the 6.3‐kb band compared with other plants, and is a *P*_*1*_ *P*_*1*_ homozygote. The pin plants in lanes 2 and 3 are labelled as *P*_*2*_ *P*_*?*_ as it is not possible to determine whether the 6.3‐kb band derives from the *P*_*1*_ or *P*_*2*_ allele. Thrum plants have a thrum‐specific 11.5‐kb band. We assigned *P*_*1*_ and *P*_*2*_ to the different pin alleles of *PvSLL1*,* PvSLL2* and *PvGlo* before genetic analysis to determine their recombination relationships to the *S* locus and each other.

The data presented in Fig. 1 define the different alleles used in our genotyping study. We extended these analyses by PCR and DNA gel blot analysis to monitor the segregation of the four *S‐linked* markers with a larger number of F_2_ *P. vulgaris* cv Blue Jeans’ progeny. Between 144 and 193 plants were used in each assay, as shown in Table 1. We did not detect recombinants between the *S* locus and *PvSLL1*,* PvSLP1* or *PvGlo* in any of the F_2_ progeny tested. However, we did observe two recombination events between the *S* locus and *PvSLL2*. The progeny numbers are small, but enabled us to determine minimum map distances based on the absence of recombination (Table 1).

**Table 1:** Summary recombination data for *S*‐linked markers in *Primula vulgaris* cv Blue Jeans

*S‐*linked marker	Pin plants	Thrum plants	Total assayed	Recombinants observed	Map distance (cM)
*PvSLP1*	92	99	191	0	< 0.52
*PvSLL1*	74	100	174	0	< 0.57
*PvSLL2*	56	90	146	2	1.37
*PvGlo*	64	93	157	0	< 0.64

Where no recombinants were found, the map distance is the theoretical maximum value based on one hypothetical recombinant in the population.

### Classical genetic analysis of the *S* locus using three‐point crosses

**Figure 2:**
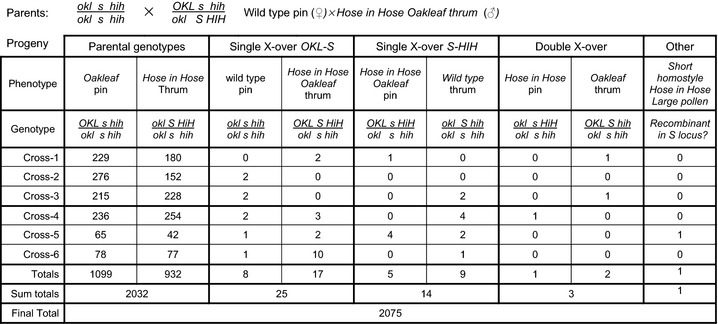
Three‐point cross to define gene order for the *S* locus, *Primula vulgaris Oakleaf* and *Hose in Hose*. Analysis of a three‐point cross between pin female parents (*s/s*), homozygous for wild‐type recessive alleles of *oakleaf* (*okl*) and *hose in hose* (*hih*), and two thrum (*S/s*) male parents, heterozygous for the dominant *Oakleaf* (*OKL*) and *Hose in Hose* (*HIH*) alleles, with *HIH* in coupling to *S* and *HIH* in repulsion to *OAK*. Phenotypes and genotypes of the different progeny classes are indicated with progeny numbers from each of six crosses shown. Crosses 1, 2 and 3 used one thrum male parent and crosses 4, 5 and 6 used a second thrum male parent. Each cross had a different pin mother. The number in each class (Totals) and numbers in each recombination category (Sum totals) are indicated. A total of 2075 progeny were characterized.

We initiated classical genetic analyses using the *S*‐linked phenotypes *Hose in Hose* (Webster & Grant, 1990; Li *et al*., 2010), *sepaloid* (Webster, 2005; Li *et al*., 2008) and *Oakleaf* (Webster, 2005; Cocker *et al*., 2015) to determine linkage relationships through two three‐point crosses. The first crossed wild‐type pin plants with thrum *Hose in Hose Oakleaf* plants with *Hose in Hose* in coupling and *Oakleaf* in repulsion to the dominant *S* allele (Fig. 2). Six crosses were established using two *Hose in Hose Oakleaf* thrum parents and five wild‐type pin parents; these crosses yielded 2075 progeny which revealed single‐ and double‐crossover events (Fig. 2). Progeny numbers were pooled to determine gene order. From these combined data, we defined the smallest progeny group, *Hose in Hose* pin and *Oakleaf* thrum plants, as double‐crossover progeny; this defines the gene order as *Oakleaf*–*S* locus–*Hose in Hose*. A further unexpected progeny class was represented by a single self‐fertile short homostyle *Hose in H*ose plant with large pollen.

**Figure 3:**
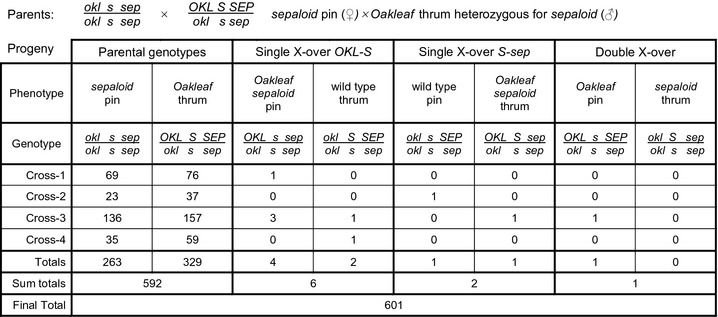
Three‐point cross to define gene order for the *S* locus, *Primula vulgaris Oakleaf* and *sepaloid*. Progeny analysis of a three‐point cross between pin female parents (*s/s*), homozygous for the wild‐type recessive alleles of *oakleaf* (*okl*) and *sepaloid* (*sep*), and thrum (*S/s*) male plants, heterozygous for the dominant *Oakleaf* (*OKL*) and recessive *sepaloid* (*sep*) alleles, with *OAK* in coupling with *S* and *sep* in coupling with *s*. Phenotypes and genotypes of different progeny classes are indicated with progeny numbers from each of four crosses shown. The number in each class (Totals) and numbers in each recombination category (Sum totals) are indicated. A total of 601 progeny were characterized.

To determine the gene order between *Oakleaf*, the *S* locus and *sepaloid*, we used a three‐point cross with a pin *sepaloid* as the female parent and four different *Oakleaf* thrums carrying a recessive *sepaloid* allele in repulsion to the dominant *S* allele, which was in coupling to *Oakleaf* (Fig. 3); these crosses yielded 601 progeny, and pooled progeny numbers were used to define the gene order. *Oakleaf* thrums and *sepaloid* pins represent nonrecombinant parental genotypes. The six *Oakleaf sepaloid* pin and wild‐type thrum individuals represent single‐crossovers between *Oakleaf* and *S*. The progeny groups of wild‐type pins and *Oakleaf sepaloid* thrums comprised two individuals; the *Oakleaf* pin and *sepaloid* thrum progeny group contained a single individual. Based on these numbers, we assigned *Oakleaf* pin and *sepaloid* thrum as the double‐crossovers; this gave a gene order of *Oakleaf*–*S* locus–*sepaloid*. These data place *Hose in Hose* and *sepaloid* on the same side of the *S* locus, opposite *Oakleaf*.

We predicted the gene order from these crosses from the combined progeny number. However, for the determination of map distances, we analysed recombinants from individual heterozygous parents separately as the recombination frequency is genotype dependent. For the *Oakleaf*–*S* locus–*Hose in Hose* cross, we used five wild‐type pin plants and two *Oakleaf*,* Hose in Hose* thrum plants in six crosses, as described previously (Fig. 2). As recombination events are only evident in chromosomes from the heterozygous thrum parents, we combined mapping data into two groups arising from crosses involving thrum‐1 and thrum‐2 (Table 2; Fig. 2); recombination in the wild‐type pin parent has no impact on progeny class and can therefore be pooled. Meiotic recombination events in thrum‐1 yielded eight *Oakleaf*–*S* locus recombinants and six *S* locus–*Hose in Hose* recombinants. Meiotic recombination events in thrum‐2 yielded 20 *Oakleaf*–*S* locus recombinants and 12 *S* locus–*Hose in Hose* recombinants (Table 2). These data give map distances of 0.62–2.55 cM between *Oakleaf* and the *S* locus and 0.39–1.53 cM between the *S* locus and *Hose in Hose* for the two pools. In both cases, we observed negative crossover interference (Auger & Sheridan, 2001) with a higher than anticipated occurrence of double‐crossovers; recombination in thrum‐1 gave a coefficient of coincidence of 103.8 and in thrum‐2 of 8.6 to give negative interference values of −102.8 and −7.6, respectively.

**Table 2:** Three‐point cross between the *Primula vulgaris Oakleaf* (*OKL*), *S* locus and *Hose in Hose* (*HIH*)

Mapping *OKL–S–HIH*	*♀* parent	*♂* parent	Total progeny	*OKL* to *S* recombinants	Distance (cM)	*S* to *HIH* recombinants	Distance (cM)	Short homostyle	Distance (cM)
Cross 1	Pin‐1	Thrum‐1	413	3	–	2	–	0	–
Cross 2	Pin‐2	Thrum‐1	430	2	–	0	–	0	–
Cross 3	Thrum‐1	Pin‐3	448	3	–	3	–	0	–
Totals from Cross 1–3			1291	8	0.62	6	0.39	0	0.00
Cross 4	Pin‐4	Thrum‐2	500	6	–	5	–	0	–
Cross 5	Pin‐5	Thrum‐2	116	3	–	6	–	1	–
Cross 6	Thrum‐2	Pin‐4	167	11	–	1	–	0	–
Totals from Cross 4–6			784	20	2.55	12	1.53	1	0.13
Combined data			2075	28	0.62–2.55	17	0.39–1.53	1	0.05

Similar analyses of the *Oakleaf*–*S* locus–*sepaloid* three‐point cross provided mapping data for the *S* locus. The four crosses that contributed to the determination of gene order (Fig. 3) used one pin *sepaloid* pollen recipient and four different heterozygous *Oakleaf* thrum plants carrying a recessive *sepaloid* allele as pollen donor. We calculated the map distances for each of the four crosses, which provided a range of 0.68–1.67 cM between *Oakleaf* and the *S* locus, and 0.67–1.64 cM between the *S* locus and *sepaloid* (Table 3). Again, we observed negative interference in Cross 3, with a coefficient of coincidence of 50.1, producing a negative interference of −49.1.

**Table 3:** Three‐point cross between the *Primula vulgaris Oakleaf* (*OKL*), the *S* locus and sepaloid (*sep*)

Mapping *OKL–S–sep*	*♀* parent	*♂* parent	Total progeny	*OKL* to *S* recombinants	Distance (cM)	*S* to *sep* recombinants	Distance (cM)
Cross 1	Pin‐1	Thrum‐1	146	1	0.68	0	< 0.68
Cross 2	Pin‐1	Thrum‐2	61	0	<1.64	1	1.64
Cross 3	Pin‐1	Thrum‐3	299	5	1.67	2	0.67
Cross 4	Pin‐1	Thrum‐4	95	1	1.05	0	< 1.05
Combined data			601	7	0.68–1.67	3	0.67–1.64

### Integrated mapping using *S* locus recombinants and molecular markers

Analysis of recombination rates by PCR or RFLP analysis with the *S* locus‐linked markers (Table 1) was limited by the logistics of screening larger numbers of plants. We therefore capitalized on recombinants from *Oakleaf*–*S* locus–*Hose in Hose* three‐point crosses. DNA was extracted from 29 *Oakleaf* to *S* locus and *S* locus to *Hose in Hose* recombinant progeny (Fig. 2). Recombinants were examined by gel blot analysis using *PvSLL1* and *PvSLL2* to determine their location with respect to *Oakleaf*,* Hose in Hose* and *S*. If all progeny resulting from the recombination of *Oakleaf* or *Hose in Hose* also showed recombination for *PvSLL1* and/or *PvSLL2*, this would place these markers outside *Oakleaf* or *Hose in Hose* with respect to *S*. If recombination of *Oakleaf* or *Hose in Hose* also leads to recombination of *PvSLL1* and/or *PvSLL2* in all cases, this would place these genes between *S* and the phenotypic markers.

**Figure 4:**
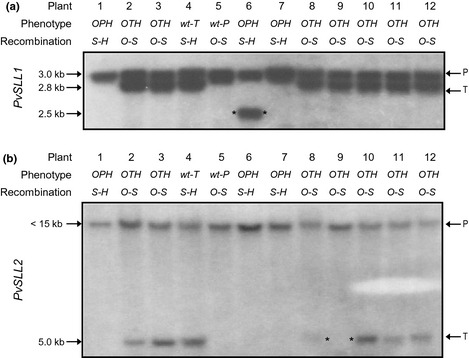
Mapping *Primula vulgaris S* locus‐linked genes onto three‐point cross recombinants. Twelve plants (1–12) obtained from the *Oakleaf*–*S* locus–*Hose in Hose* three‐point cross, that represent single‐crossover recombination events between either *Oakleaf* and the *S* locus or *Hose in Hose* and the *S* locus, are shown. The phenotypes of individuals (*O*,* Oakleaf*;* P*, pin; *T*, thrum; *H*,* Hose in Hose*; wt, wild‐type) are indicated, as well as the location of the crossover in each case in relation to the *S* locus (*S*) and the phenotypic markers *Oakleaf* (*O*) and *Hose in Hose* (*H*). (a) Autoradiograph of gel blot analysis of *Hind* III‐digested genomic DNA using *PvSLL1* as probe. Pin (P)‐ and thrum (T)‐specific alleles of *PvSLL1*, as revealed by restriction fragment length polymorphisms (RFLPs), are indicated. An inconsistent RFLP obtained from plant 6 is identified by asterisks. (b) Autoradiograph of gel blot analysis of *Xba*I‐digested genomic DNA using *PvSLL2* as probe. P‐ and T‐specific alleles of *PvSLL1*, as revealed by RFLPs, are indicated. The absence of a thrum‐specific RFLP in plant 9 is identified by asterisks.

Eighteen of the 25 *Oakleaf*–*S* recombinants and 11 of the 14 *Hose in Hose*–*S* recombinants (Fig. 2) were analysed by gel blot analysis. Data from 12 representative plants, four recombinants between the *S* locus and *Hose in Hose* (*S–H*) and eight from recombination between *Oakleaf* and the *S* locus (*O–S*), are shown in Fig. 4. The *PvSLL1* allele in coupling with the *s* allele is represented by a 3.0‐kb RFLP (Fig. 4a); the thrum allele*,* in coupling to the *S* allele, is represented by a 2.8‐kb RFLP. None of the eight plants arising from recombination between *Oakleaf* and *S* show assortment for *PvSLL1*; all are thrum and heterozygous for 3.0‐kb pin and 2.8‐kb thrum alleles of *PvSLL1*. These data show that *Oakleaf* has recombined independently of *PvSLL1*;* PvSLL1* is therefore not distal to *Oakleaf* with respect to the *S* locus. Three of the four progeny showing recombination between the *S* locus and *Hose in Hose* (plants 1, 4 and 7) also show that recombination of *Hose in Hose* does not affect *PvSLL1*. The two pin plants (plants 1 and 7) are homozygous for the 3.0‐kb pin allele of *PvSLL1*, and the thrum plant (plant 4) is heterozygous for the pin and thrum alleles of *PvSLL1*. *PvSLL1* cannot therefore be outside *Hose in Hose* with respect to the *S* locus. Interestingly, plant 6 reveals a 2.5‐kb *PvSLL1* allele in addition to the 3.0‐kb pin allele. This band either represents a deletion or a point mutation that affects the RFLP, or a recombination event between the *S* locus and *PvSLL1* that affects the size of the RFLP. If this band represents a recombination event, this places *PvSLL1* between the *S* locus and *Hose in Hose*. Subsequent analysis of the BAC contig spanning the *S* locus confirmed this location (see later).

Similarly, *PvSLL2* is represented in this population by two alleles, a < 15‐kb band in coupling to the *s* allele, and a 5.0‐kb band in coupling with the *S* allele (Fig. 4b). None of the four plants showing recombination between *S* and *Hose in Hose* show assortment of *PvSLL2* alleles. *PvSLL2* cannot therefore be distal to *Hose in Hose* with respect to the *S* locus. Of the eight plants shown resulting from recombination between *Oakleaf* and *S*, seven show heterozygosity for the two *PvSLL2* alleles. Recombination of *Oakleaf* from the *s* chromosome onto the *S* chromosome does not consistently bring with it the < 15‐kb pin allele of *PvSLL2*. This marker is therefore not distal to *Oakleaf* with respect to the *S* locus. However, one *Oakleaf*–*Thrum*–*Hose in Hose* recombinant, plant 9, is homozygous for the < 15‐kb pin allele of *PvSLL2* and lacks the 5.0‐kb thrum allele. This individual represents a recombination event between *PvSLL2* and the *S* locus, and places *PvSLL2* between *Oakleaf* and the *S* locus. None of the other 17 plants analysed by Southern analysis (data not shown) showed recombination between either *PvSLL1* and *S* or *PvSLL2* and *S*. Only 28 plants showing recombination between *Oakleaf* and *S* were identified in 2075 progeny; of these, only one had recombined for *PvSLL2*. Based on these data, we assign a map distance between *PvSLL2* and the *S* locus of 0.05 cM. Similarly, if the 2.5‐kb *PvSLL1* RFLP in plant 6 (Fig. 4) arose by recombination, this would produce a map distance of 0.05 cM between *PvSLL1* and the *S* locus (Table 4). It remains possible that this 2.5‐kb band represents a mutation rather than recombination; if this is the case, the map distance must be < 0.05 cM. As discussed below, the sequence of the BAC contig surrounding the *S* locus confirms that *PvSLL1* is located between the *S* locus and *Hose in Hose*.

**Table 4:** Recombinants for *PvSLL1* and *PvSLL2* mapped onto *Primula vulgaris* three‐point cross recombinants

*OKL–S–HIH three‐point cross*	*PvSLL1*	*PvSLL2*
Total progeny	2075	2075
*OKL* to *S* single‐crossover events	28	–
Recombinants analysed by blot	18	–
*S*–*PvSLL1* recombinants	1^a^	–
Map distance (cM)	0.05	–
*S* to *HiH* single‐crossover events	–	17
Recombinants analysed by blot	–	11
*S*–*PvSLL2* recombinants	–	1
Map distance (cM)	–	0.05

**Figure 5:**
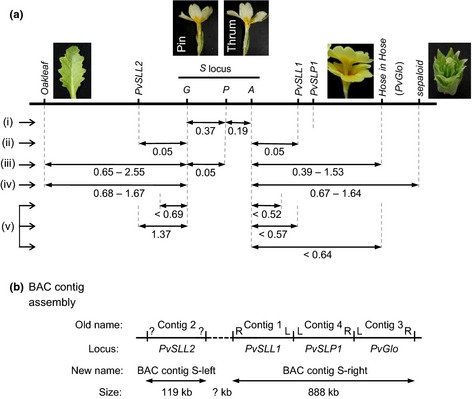
A genetic map of the *Primula vulgaris S* locus. (a) The relative positions of the mutants *Oakleaf*,* Hose in Hose* and *sepaloid* are indicated in relation to the *S* locus and its constituent genes *G*,* P* and *A*. The mapped locations of *PvSLL1 and PVSLL2* and *PvSLP1* and *PvSLP2* are indicated. Two potential locations for *PvSLP2* (light grey) are shown, as the precise map position is not defined. Map distances are shown in cM and were determined by: (i) Lewis & Jones (1993); (ii) data from Fig. 4 and Table 4; (iii) data from Fig. 2 and Table 2; (iv) data from Fig. 4 and Table 3; (v) data from Table 1. Images of phenotypic markers are included next to the relevant loci. (b) Summary of BAC contig assembly results. The resolved order and current assembly of the four BAC assembly contigs described previously (Li *et al*., 2011) are indicated (Old name), and their orientation is given by L and R, or ‘?’ if unknown. The *S* locus‐linked marker associated with each of the four previous BAC contigs is indicated. The revised BAC assembly contig name (New name) is indicated, together with the size estimated from the assembly. The region with no BAC coverage is shown as a dotted line.

*HiH*,* Hose in Hose*;* OKL*,* Oakleaf*;* S*,* S* locus.

a Plant identified in Fig. 5(a) that gave an aberrant restriction fragment length polymorphism (RFLP) profile and may represent a recombinant.

Data from the three‐point crosses, and analysis of assortment of pin and thrum alleles for molecular markers, generated a map of the *S* locus (Fig. 5a) which includes the order and relative position of the mutants, *Oakleaf* (Webster, 2005; Cocker *et al*., 2015), *Hose in Hose* (*PvGlo*) (Li *et al*., 2010) and *sepaloid* (Li *et al*., 2008), alongside three molecular markers, *PvSLL1* (Webster & Grant, 1990; Li *et al*., 2007), *PvSLL2* (Li *et al*., 2007) and *PvSLP1* (Manfield *et al*., 2005). *PvSLP1* was identified as a RAPD marker specific to *P. vulgaris* Blue Jeans, and so we could not analyse its segregation in these crosses. However, BAC contig assembly and sequencing (see later) defined the location of *PvSLP1* on the map (Fig. 5b).

### Localization of the *S* locus by chromosome *in situ* hybridization

**Figure 6:**
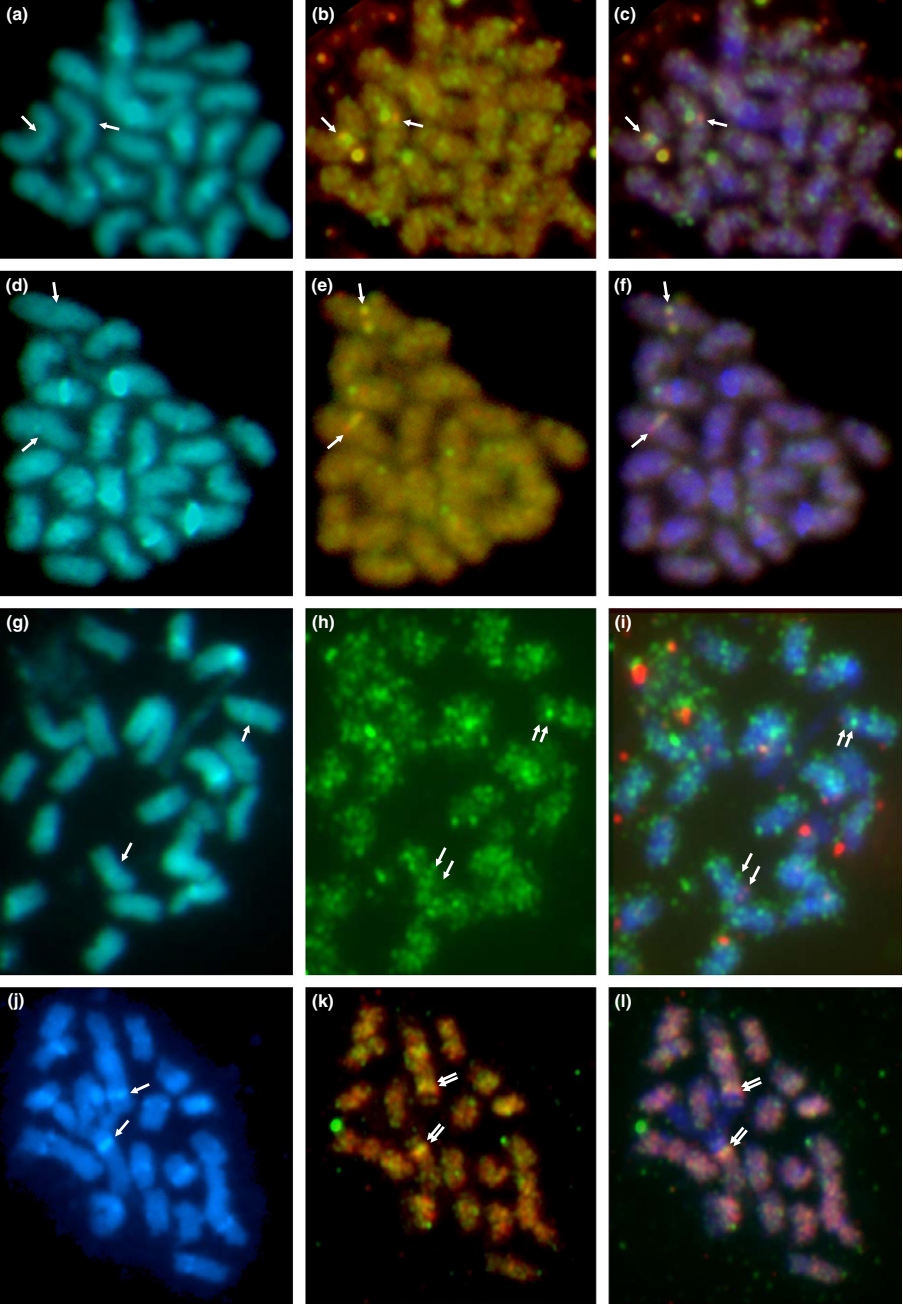
Chromosome *in situ* hybridization reveals the location of the *Primula S* locus. Hybridization to metaphase chromosomes of *P. vulgaris* (2*n *= 22; fluorescing blue with 4′,6‐diamidino‐2‐phenylindole, DAPI) using S locus‐linked BACs. (a–f) BAC56H19 (detected by red fluorescence) and BAC81I19 (green fluorescence). (g–i) BAC56H19 (red) and BAC13A4 (green) showing colocalization (yellow–orange when overlaid). (j–l) BAC13A4 (red) and BAC81B15 (green). (a, d, g, j) Chromosomes with intercalary DAPI‐positive bands on some chromosomes, and less diffuse labelling in the terminal region, suggesting differentiation of repeat content along the chromosome. (b, e, h, k) Hybridization with *S* locus‐linked BACs detected by red and green fluorescence; overlapping signals are yellow–orange or green–purple depending on the intensity of the fluorochromes. Arrows indicate: (a, d, g, j) centromeric regions; (b, c, e, f) superimposed fluorescence signals; (h, i, k, l) adjacent fluorescence signals. (c, f, i, l) Overlapping probe and DAPI fluorescence images. In (h, i, k, l), the green signal is proximal to the centromere.

Previous cytogenetic studies have suggested that the *S* locus is located close to the centromere in some species of *Primula* (Darlington, 1931; Dowrick, 1956; Lewis & Jones, 1993). The availability of BAC clones from sequences flanking the *S* locus has enabled us to directly visualize the location. Initially, we used two overlapping BACs (BAC56H19 (red) and BAC81I19 (green)) (Fig. 6a–f) from the *PvSLL2* side of the *S* locus (Fig. S1) for chromosome *in situ* hybridization on metaphase chromosomes. The 22 chromosomes were visualized with DAPI (Fig. 6a,d). A merged DAPI–fluorescence image (Fig. 6c,f) reveals the location of the *S* locus as centromeric on the largest metacentric chromosome pair. Data from two independent experiments are shown (Fig. 6a–f).

A second analysis using BAC56H19 (red) and BAC13A4 (green), which map to either side of the *S* locus (Fig. S1), is shown in Fig. 6g–i. The signal obtained from BAC13A4 is quite diffuse (Fig. 6h); however, as indicated by the arrows (Fig. 6i), these probes colocalize. Comparison with DAPI‐stained chromosomes (Fig. 6g) reveals that BAC56H19 is distal and BAC13A4 is proximal to the centromere. We also analysed BAC13A4 (red) and BAC81B15 (green), which are both on the *PvGlo* side of the *S* locus (Fig. S1). These data (Fig. 6j–l) show that BAC81B15 is closer than BAC13A4 to the centromere, and support the observations with BAC56H19 and BAC13A4 (Fig 6i) that the *PvGlo* side of the *S* locus is proximal to the centromere.

### Assembly and sequencing of BAC contigs surrounding the *S* locus

We have described previously the construction of two *P. vulgaris* BAC libraries and their screening using four *S* locus‐linked probes, *PvSLL1* and *PvSLL2* (Li *et al*., 2007), *PvSLP1* (Manfield *et al*., 2005) and *PvGlo* (Li *et al*., 2010), and the assembly of four BAC contigs around these probes (Li *et al*., 2011). We have extended and completed this assembly by further BAC‐end screening and walking. These analyses identified several new BACs which clarified existing ambiguities in the contig assemblies, and provided new sequence to bridge gaps in the earlier assembly. The order and integration of the previous four contigs (Li *et al*., 2011) is shown in Fig. 5(b). Contig 3 sits to the right of the map; our new data show that the left‐hand end of contig 3 overlaps with the right‐hand end of contig 4, and the left‐hand end of contig 4 now links to the left‐hand end of contig 1 (Fig. 5b). The new BAC clones which facilitated the assembly and enabled the integration of three contigs into one are shown in bold and identified by plus signs in Fig. S1. This integrated contig covers 888 kb and was renamed *Contig S‐right* (Fig. 5b). This contig links, and provides a gene order for, *PvSLL1*,* PvSLP1* and *PvGlo* (Fig. S1). We have not been able to join contig 3 (Li *et al*., 2011), renamed *Contig S‐left*, to the larger assembly (Fig. 5b). BAC‐end sequences from the right‐hand end of contig 1 and both ends of contig 2 are highly repetitive and identified multiple BACs in subsequent screening rounds. We have aligned the assembled BAC contigs to our genetic map based on the location of *PvSLL1*,* PvSLL2*,* PvSLP1* and *PvGlo* to integrate genetic and physical maps (Fig. 5a). These data are summarized in Fig. 5(b) and expanded in detail in Fig. S1.

The sequencing of 10 individual BACs from across the contig assemblies enabled us to assemble sequence contigs associated with the BAC contigs. We also sequenced two pools of BACs. Five BACs sequenced individually were also included in the pools (Fig. S1). Pool 1 contained 19 BACs from contigs 1 and 2 (Li *et al*., 2011), and pool 2 contained 18 BACs from contigs 3 and 4 (Li *et al*., 2011); the relative positions of these contigs are shown in Fig. 5 and are fully expanded in Fig. S1. The assembly of individually sequenced BACs, together with sequence from pooled BACs, generated four sets of sequence contigs. Group‐A: overlapping DNA sequence anchored to known BACs from *Contig S‐left* yielded 17 contigs covering 119 kb (Fig. S1). Group‐B: overlapping DNA sequence anchored to BACs from *Contig S‐right* yielded 58 contigs covering 888 kb. Group‐C: unanchored sequence contigs from pool 1 BACs comprised 56 contigs covering 325 kb. Group‐D: unanchored sequence contigs from pool 2 comprised 99 contigs covering 178 kb. In total, 1.5 Mb of sequence flanking the *S* locus was assembled; 1 Mb is anchored to specific BACs (Fig. S1). It was not possible to generate a contiguous assembly across the entire region, and this is reflected by the assembly gaps in Group‐A (17 contigs, 16 gaps) and Group‐B (58 contigs, 57 gaps), and the unanchored contigs in Group‐C and Group‐D. The 17 contigs in Group‐A (*Contig S‐left*) and 58 contigs in Group‐B (*Contig S‐right*) were ordered relative to the BAC tiling path (Fig. S1); contigs residing between BAC‐ends could not be oriented or ordered relative to each other. Although it is not possible to assign an unambiguous order for all intervening contigs from specific BACs, a high level order of these contigs is shown in Table S1. Contigs containing BAC‐ends are highlighted in bold. The order of intervening contigs remains to be confirmed.

### Gene annotation within the BAC contigs flanking the *S* locus

We undertook *de novo* annotation of BAC sequence contigs (Fig. S1; Table S1). The number of predicted genes and gene fragments in each of the four BAC sequence groups is summarized in Table S2; 266 potential genes or gene fragments were identified. Of these sequences, 119 identified known proteins in BlastX searches; the remaining 147 predicted gene fragments gave no database similarities. We searched the *Arabidopsis* TAIR10 database and, after removal of duplicates caused by gene models on the same contig matching the same *Arabidopsis* gene, or the same *Arabidopsis* locus matching gene predictions on neighbouring contigs, we found 82 related *Arabidopsis* genes.

Annotation data for genes associated with *Contig S‐left* and *Contig S‐right* are presented in Table S2. Confirmation of gene order on internal BAC contigs remains to be confirmed. Within these contigs, we identified *PvSLL1* on S_locus_groupB_ctg13 and *PvGlo* on S_locus_groupB_ctg58; we also located *PvSLP1* to S_locus_groupB_ctg36. These data confirm the order of *S*‐linked markers on BAC contig assemblies (Fig. 5) and unequivocally demonstrate the order as, *S* locus–*PvSLL1–PvSLP1–PvGlo,* within 888 kb of assembled sequence. We also identified *PvSLL2* on contig S_locus_groupA_ctg9 within *Contig S‐left*. The full annotation and orientation of predicted gene models in the assembled 1.5‐Mb sequence will require manual curation and integration with the *P. vulgaris* genome sequence.

## Discussion

*Primula* provides one of the earliest examples, after Mendel's peas (Bateson, 1902; Bhattacharyya *et al*., 1990), of a model for genetic analysis. Bateson & Gregory (1905) revealed the dominance relationship of pin and thrum flowers which led to the identification of *S*‐linked phenotypes in *P. sinensis* (Gregory *et al*., 1923; De Winton & Haldane, 1935). This work provided one of the first examples of linkage in plants and one of the first linkage maps (Gregory, 1911; Bridges, 1914; Altenburg, 1916). Yoshida *et al*. (2011) recently took a different approach and used QTL analysis to develop a genome‐wide linkage map that identified *S* locus‐linked markers in *P. sieboldii*.

We pursued a classical approach to generate a genetic map for *P. vulgaris* and used *Hose in Hose* (Ernst, 1942; Webster & Grant, 1990; Li *et al*., 2010) with two other *S* locus‐linked mutant phenotypes*, sepaloid* (Webster, 2005; Li *et al*., 2008) and *Oakleaf* (Webster, 2005; Cocker *et al*., 2015), together with the heterostyly phenotypes, to generate a linkage map. We have not found phenotypes corresponding to the *P. sinensis S*‐linked loci, *magenta, red stigma*,* red leaf back* or *double* (De Winton, 1928; De Winton & Haldane, 1935), in *P. vulgaris*.

Three‐point cross analysis enabled us to establish a gene order of *Oakleaf*–*S* locus–*Hose in Hose*, with *sepaloid* predicted to be on the same side as *Hose in Hose* (Figs 3, 4). Map distances for *Oakleaf* to *S* range from 0.62 to 2.55 cM, and for *S* to *Hose in Hose* from 0.39 to 1.53 cM. This range reflects different recombination rates in different individual parents. Given the limited number of phenotypic markers, we were fortunate to find that *Oakleaf* and *Hose in Hose* mapped to either side of the *S* locus (Fig. 2). The three‐point cross involving *sepaloid* involved fewer progeny (Fig. 3); assignment of the double recombinants was therefore less obvious than for the *Hose in Hose* cross. However, our data indicate that *sepaloid* is on the same side of the *S* locus as *Hose in Hose,* with a gene order of *Oakleaf*–*S* locus–*sepaloid*; the map distance ranges from 0.68 to 1.67 cM for *Oakleaf* to *S*, and from 0.67 to 1.64 cM for *S* to *sepaloid*. These combined data enabled us to establish a genetic map with phenotypic markers flanking the *S* locus (Fig. 5). In both three‐point crosses, we observed negative interference, revealed by a higher than expected rate of double recombinants. It is interesting to note that de Winton and coworkers reported differences in male and female recombination rates in *P. sinensis* (Gregory *et al*., 1923; De Winton & Haldane, 1933, 1935). Like ours, their data also came from different crosses, and it may be that different recombination rates for male and female parents simply reflect differences between individuals, as seen in our data (Table 2), rather than gender differences.

Our previous studies defined *S* locus linkage of four sequences (Fig. 1) by RFLP and PCR analysis using modest numbers of plants (Manfield *et al*., 2005; Li *et al*., 2008, 2010). We also previously defined *Hose in Hose* as a mutation in *PvGlo* (Li *et al*., 2010). Combined data from these different studies are integrated in Fig. 1 and Table 1. We therefore took advantage of the recombinants from large three‐point crosses segregating for *Oakleaf*,* Hose in Hose* and heterostyly to increase the resolution of these map distances and position the markers with respect to phenotypic markers. We were again fortunate that our sequence markers mapped to either side of the *S* locus (Fig. 5). The RAPD marker *PvSLP1* was identified in *P. vulgaris* Blue Jeans, but is not detectable in all cultivars. Therefore, we could not map *PvSLP1* in three‐point cross progeny. However, sequence analysis of the *S* locus BAC contigs provides an unambiguous location for *PvSLP1* between *PvSLL1* and *PvGlo* (Fig. S1; Tables S1, S2), as summarized in Fig. 5.

The appearance of a self‐fertile short homostyle in a mapping cross (Fig. 2) was a surprise. Although Ernst (1925, 1933, 1936b) identified and characterized homostyles in *P. viscose* and *P. hortensis*, De Winton & Haldane (1935) did not find any homostyles in 18 000 plants during their genetic studies of *P. sinensis*. Dowrick's analysis of diploid *P. obconica* did not identify any homostyles in 5000 plants (Dowrick, 1956), and neither did Ernst (1928b) in 8000 plants studied. Homostyles are therefore very rare. They are known in *P. vulgaris* and were noted by Darwin (1877), and have since been studied in populations in Somerset (Bodmer, 1960; Piper *et al*., 1984; Curtis & Curtis, 1985) and the Chilterns (Crosby, 1940, 1948). Both of these populations contain only long homostyles. Ernst (1928b) originally considered mutations to be responsible for the breakdown of heterostyly, but Dowrick (1956) and Lewis & Jones (1993) interpreted homostyles as resulting from recombination within the *S* locus that disrupts the coupling of dominant alleles within the *S* locus, and also leads to the breakdown of the self‐incompatibility system to generate self‐fertile homostyles.

It is possible that low levels of recombination within the *S* locus could reflect a genome rearrangement between the two alleles, for example an inversion (De Winton & Haldane, 1935; Mather, 1950), or may be a consequence of proximity to the centromere (Dowrick, 1956). Evidence from double reduction (Darlington, 1931) in tetraploid plants suggests that the *S* locus is located close to the centromere in *P. sinensis* (De Winton & Haldane, 1935) and *P. obconica* (Dowrick, 1956). We have shown directly by double‐labelling chromosome *in situ* hybridization using overlapping BACs (Fig. 6) that the *S* locus in *P. vulgaris* is located close to the centromere of the largest metacentric chromosome. This direct visualization confirms the prediction made over 80 yr ago by cytogenetic analysis (Darlington, 1931). Darlington (1931) also speculated that the chromosome carrying the *S* locus in *P. sinensis* might be the largest, as it carried the greatest number of *S*‐linked loci; again, our chromosome *in situ* data confirm his prediction. Our *in situ* analyses also orientate the *S* locus map with respect to the centromere, and show that the *PvGlo* side of the *S* locus is proximal to the centromere (Fig. 6).

The assembly of over 1 Mb of sequence anchored to BACs surrounding the *S* locus (Fig. S1; Table S1) has identified at least 82 new *S* locus‐linked genes (Table S2); a further 500 kb of *S*‐linked sequences remain to be anchored, and this will be facilitated with a more complete *Primula* genome assembly. Although we cannot yet predict the size of the gap between the two contigs, our data suggest that *PvSLL2* and *PvGlo* are at least 1.5 Mb apart. *PvGlo* is at least 888 kb from the *S* locus (Fig. S1; Table S2), and *Hose in Hose* (*PvGlo*) is between 0.39 and 1.53 cM from the *S* locus; this suggests a relationship of 1 cM to 580–2277 kb.

It remains to be seen whether the assembled BAC contigs include the key *S* locus genes or whether these reside within the gap between contigs. *PvSLL2* is located within a contig flanked by 66.6 and 27.1 kb on either side (Table S1). None of the predicted genes in this contig are obvious candidates for *Oakleaf* (Webster, 2005; Cocker *et al*., 2015), and none of the genes upregulated in *Oakleaf* (Cocker *et al*., 2015) are found in this contig. This is perhaps not surprising as *PvSLL2* is only 0.05 cM from the *S* locus, and *Oakleaf* is between 0.56 and 2.55 cM away (Fig. 5). The identification of *Oakleaf* will require the analysis of the full genome sequences and demonstration of linkage between candidate genes and the *S* locus. The *de novo* annotation of the BAC assembly identified 266 gene models, 82 of which correspond to known *Arabidopsis* genes (Table S2); ongoing annotation of the *P. vulgaris* genome and transcript‐driven gene model definition will resolve the number of genes. We have not found sequences corresponding to those identified in *F. esculentum* and *L. grandiflorum* in our gene annotations (Table S2), and this possibly reflects the polyphyletic origins for floral heteromorphy.

*Primula* has a long history as a model for studies on floral heteromorphy, built on Darwin's landmark studies (Darwin, 1862) and the various historical observations on heterostyly before his work (van Dijk, 1943; P. M. Gilmartin, unpublished); this plant has also played a fundamental role in the establishment of Mendelian genetic analysis through the attention of Bateson, Haldane, Bridges (Bateson & Gregory, 1905; Bridges, 1914; Haldane, 1933) and others in the early 1900s. One hundred years later, we have generated an integrated genetic and physical map of the *P. vulgaris S* locus, localized and orientated it by *in situ* chromosome hybridization, and identified 82 new *S* locus‐linked genes. Based on this study, and our ongoing annotation of the *P. vulgaris* genome, we are poised to identify the *S* locus genes which control floral heteromorphy in *Primula*.

## Supporting information

Please note: Wiley Blackwell are not responsible for the content or functionality of any supporting information supplied by the authors. Any queries (other than missing material) should be directed to the *New Phytologist* Central Office.

**Fig. S1 ** Map of the *S* locus showing assembly of the BAC contig flanking the regions.
**Methods S1 ** Bioinformatic supplemental methods.

**Table S1 ** Sequence contig assemblies derived from *S* locus‐associated BAC sequencing
**Table S2 ** Annotation of sequence contig assemblies derived from S locus‐associated BACs
