# Supplementary material for: Integration of genetic and physical maps of the Primula vulgaris S locus and localization by chromosome in situ hybridization
Source: New Phytol. 2015 Apr 9;208(1):137–48. doi: 10.1111/nph.13373 (PMC6680154; doi:10.1111/nph.13373)
Supplement: Supplementary file 1 — Fig. S1 Map of the S locus showing assembly of the BAC contig flanking the regions. Methods S1 Bioinformatic supplemental methods. [file NPH-208-137-s001.pdf]

***New Phytologist* Supporting Information Fig. S1 and Methods S1**

Article title: Integration of genetic and physical maps of the *Primula vulgaris S* locus and localization by chromosome *in situ* hybridization

Authors: Jinhong Li, Margaret Webster, Jon Wright, Jonathan M. Cocker, Matthew C. Smith, Farah Badakshi, Pat Heslop-Harrison and Philip M. Gilmartin

Article acceptance date: 07 February 2015

The following Supporting Information is available for this article:

Fig. S1 Map of the *S* locus showing assembly of the BAC contig flanking the regions.
Table S1 Sequence contig assemblies derived from *S* locus associated BAC sequencing (separate Excel file)
Table S2 Annotation of sequence contig assemblies derived from S locus associated BACs (separate Excel file)
Methods S1 Bioinformatic supplemental methods. [Correction added after online publication 9 April 2015; three URLs in the text have been updated.]

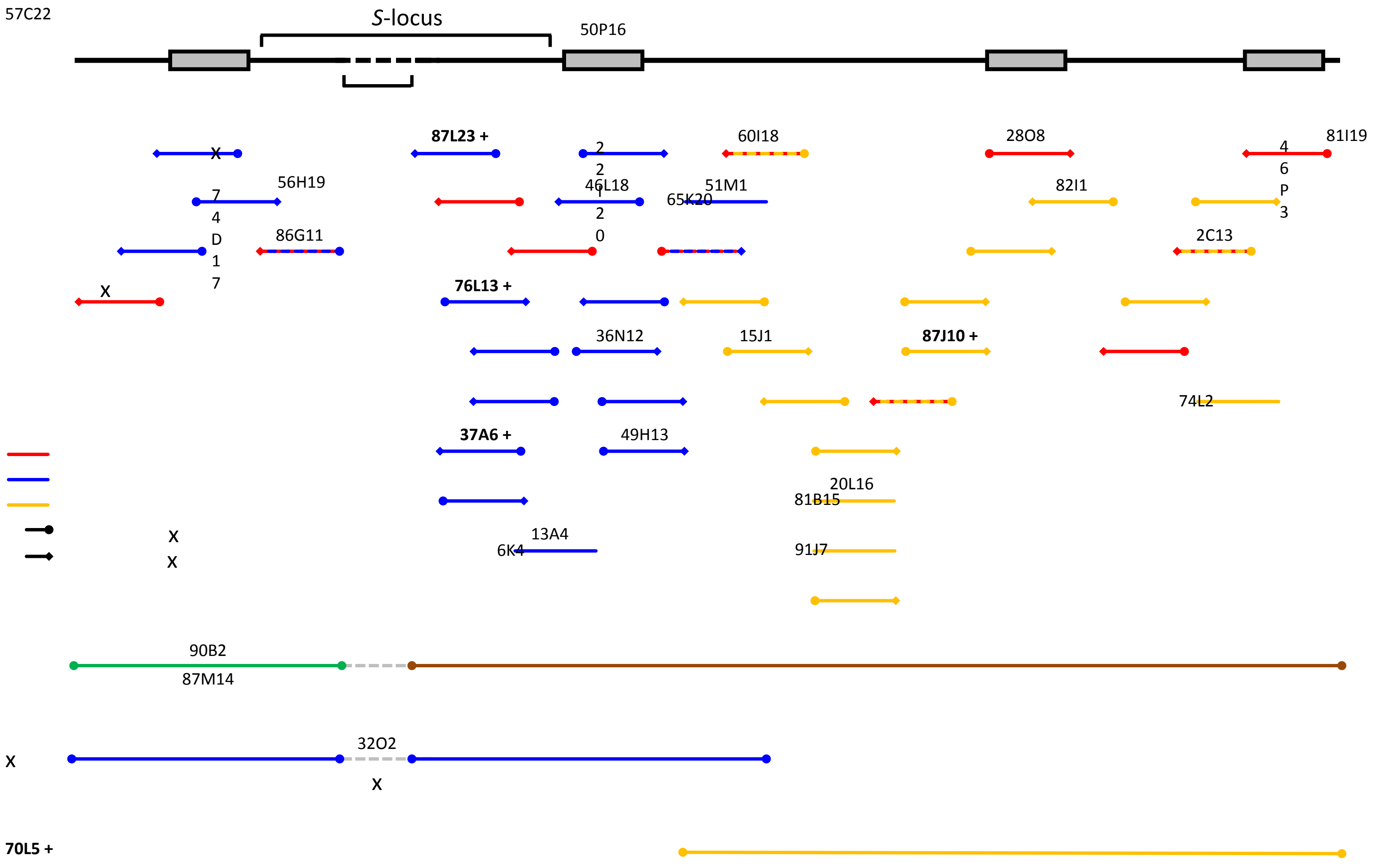

Fig. S1 Map of the *S* locus showing assembly of BAC contigs flanking the region. (a) The structure of the region flanking the *S* locus is shown. DNA markers used to map the region are shown as grey boxes, intervening sequences as a line. The *S* locus location is indicated. The region with no BAC coverage is arbitrarily shown by a dotted line; the precise location of this gap in relation to the S locus is not known so this is a diagrammatic representation. Individual BACs contributing to the contig are shown with their identify code. Those BACs which include the original sequences corresponding to one of the four DNA probes are identified by crosses; left and right BAC ends are shown by diamonds and circles, respectively. The

four BACs used for chromosome *in situ* analysis are identified by an asterisk. Those BACs which helped resolve ambiguities and link previous contigs (Li *et al.*, 2011) are indicted by plus signs. BACs sequenced individually are shown in red, those sequenced in sequence pool 1 are shown in blue, and those sequenced in pool 2 are shown in yellow. BACs sequenced individually and as part of a pool are shown in dotted lines of the two colours. (b) The region of coverage of sequence contigs derived from assembly of individual and pooled BAC sequencing reads that are anchored to BAC Contig S-left and BAC contig S-right are represented by a green line and brown line respectively and labelled as Group-A and Group-B. The number of contigs and coverage is indicated. The region with no BAC coverage is indicated by a dotted gray line. (c) The potential location of unanchored sequence contigs derived from pool 1 BAC sequence data is represented by a blue line. The number of contigs and their coverage is indicated. The region with no BAC coverage is indicated by a dotted gray line. (d) The potential location of unanchored sequence contigs derived from pool 2 BAC sequence data is represented by a yellow line. The number of contigs and their coverage is indicated.

Reference

Li J, Webster MA, Smith MC, Gilmartin PM. 2011. Floral heteromorphy in *Primula vulgaris*: progress towards isolation and characterization of the S locus. *Annals of Botany* **108:** 715–726.

Methods S1 Bioinformatic supplemental methods.

After removing *Escherichia coli* and vector contamination, 454 reads were assembled using the 454 assembler gsAssembler (v2.6); Illumina reads were assembled using ABySS (v1.3.6) generating a set of contigs for each individually sequenced BAC, and a set of contigs for each BAC pool. After including available BAC-end sequences, the clustering software cd-hit (Fu *et al.*, 2012) was used to remove redundant contigs from the set and minimus2 (Sommer *et al.*, 2007) was used to merge overlapping contigs and BAC-end sequences (sequence identity >98%). The thrum whole genome sequence paired-end reads were assembled using ABySS (v1.3.6) and scaffolded using SOAPdenovo (v2.04). Read coverage in this study from the 9 kb mate pair library was 32×; the same library used in the accompanying paper (Cocker *et al.*, 2015) provides 26× coverage due to a more stringent quality trimming strategy being applied to the raw mate-pair reads. The merged set of *S* locus contigs was used to identify whole genome sequence contigs potentially originating from the *S* locus region and these were also incorporated into the assembly using minimus2. BLAT v3.5 (Kent, 2002) was used to identify regions of overlap >500 bp between contigs and these contigs were merged to generate the final contig set. Contigs were anchored along the *S* locus by aligning BAC-end sequences, marker sequences and original BAC contigs using exonerate v2.2.0 (Slater & Birney, 2005).

RepeatModeler (http://www.repeatmasker.org/RepeatModeler.html) was used with a draft thrum genome assembly to identify *de novo* repetitive sequences; these were hard-marked for inclusions from protein coding genes via BlastX alignments (e-value 1×10^{-4}) to protein databases from *Actinidia chinensis* (http://bioinfo.bti.cornell.edu/cgi-bin/kiwi/home.cgi), *Mimulus guttatus* v2.0, *Solanum tuberosum*, and *Solanum lycopersicum* (http://phytozome.jgi.doe.gov/).

The repeat library produced comprised the hard-masked sequences which had at least one alignment to a transposition-associated domain from Pfam-A (curated thresholds) or Pfam-B (e-value 1×10^{-4}); alignments were carried out using HMMer hmmscan v3.1b1) (http://pfam.sanger.ac.uk/; http://hmmer.janelia.org/search/hmmscan). Pfam domains were considered transposition-associated if they aligned with any of the sequences contained in the database of transposable elements included in the RepeatRunner package (http://www.yandell-lab.org/software/repeatrunner.html).

Short interspersed repeats and low-complexity repetitive sequences were identified and hard-masked in the BAC assembly using the repeat library with a local installation of RepeatMasker based on the RMBlast algorithm (version open-4.0.1: http://www.repeatmasker.org/). The self-training gene annotation program GeneMark-ES (http://opal.biology.gatech.edu/) was used with the draft thrum genome assembly to produce a training file which served as an input for the *de novo* gene finder GeneMark-E in the annotation of the repeat-masked BAC assembly. The gene models obtained for each contig were scanned with the software package Full-LengtherNEXT (e-value 1×10^{-4}) (http://www.scbi.uma.es/site/scbi/downloads/313-full-lengthernext) in order to classify them as full-

length, 5'-end, 3'-end or internal. BlastX (e-value 1×10^{-4}) was used to query the putative genes against the TAIR10 *Arabidopsis thaliana* protein database (http://www.arabidopsis.org/) and the NCBI non-redundant protein database, the result of the latter being an input for further annotation with Blast2GO (https://www.blast2go.com/) (Conesa *et al.*, 2005) (see Table S2).

References

Cocker J, Webster MA, Li J, Wright J, Kaithakottil GG, Swarbreck D, Gilmartin PM. 2015. *Oakleaf:* an *S* locus-linked mutantion of *Primula vulgaris* that affects leaf and flower development. *New Phytologist.* doi: 10.1111/nph.13370.

Conesa A, Gotz S, Garcia-Gomez JM, Terol J, Talon M, Robles M. 2005. Blast2GO: a universal tool for annotation, visualization and analysis in functional genomics research. *Bioinformatics* **21:** 3674–3676.

Fu L, Niu B, Zhu Z, Wu S, Li W. 2012. CD-HIT: accelerated for clustering the next-generation sequencing data. *Bioinformatics* **28:** 3150–3152.

Kent WJ. 2002. BLAT – the BLAST-like alignment tool. *Genome Research* **12:** 656–664.

Slater GS, Birney E. 2005. Automated generation of heuristics for biological sequence comparison. *BMC Bioinformatics* **6:** 31.

Sommer DD, Delcher AL, Salzberg SL, Pop M. 2007. Minimus: a fast, lightweight genome assembler. *BMC Bioinformatics* **8:** 64.
